# Studies Using Antibodies against Filaggrin and Filaggrin 2 in Canine Normal and Atopic Skin Biopsies

**DOI:** 10.3390/ani14030478

**Published:** 2024-02-01

**Authors:** Rosanna Marsella, Kim Ahrens, Rachel Wilkes

**Affiliations:** Department of Small Animal Clinical Sciences, College of Veterinary Medicine, University of Florida, Gainesville, FL 32611, USA; sciencematters2me@gmail.com (K.A.); rachelsusansanford@gmail.com (R.W.)

**Keywords:** filaggrin, dog, atopic dermatitis

## Abstract

**Simple Summary:**

In people, skin allergies to environmental factors have been linked to abnormalities in the skin itself. One of these abnormalities in people is a lower amount of a family of skin proteins called filaggrins. This decrease makes the skin more permeable to the absorption of environmental allergens such as dust mites. In our study, we wanted to use a dog model of skin allergies to study the impact of standardized and repeated dust mite exposures on the skin itself. More specifically, we wanted to see how these exposures would affect filaggrin proteins and compare the response of normal and allergic dogs. We found that the skin of allergic dogs overreacted to dust mite exposure by getting thicker and increasing the production of filaggrins compared to the normal dogs. Despite this extra production of filaggrin, the allergic skin showed more breakdown products of filaggrin. We conclude that allergic skin attempts to compensate for the allergic insult but it is not as efficient as normal skin.

**Abstract:**

Filaggrin is important for the skin barrier and atopic dermatitis. Another filaggrin-like protein, filaggrin 2, has been described. We evaluated antibodies against both filaggrins in normal and atopic skin biopsies from dogs before and after allergen challenges (D0, D1, D3 and D10). Filaggrins expression was evaluated by immunohistochemistry and Western blot. We used PCR to investigate changes in filaggrin gene expression. Effects of group (*p* = 0.0134) and time (*p* = 0.0422) were shown for the intensity of filaggrin staining. Only an effect of group was found for filaggrin 2 (*p* = 0.0129). Atopic samples had higher intensity of staining than normal dogs [filaggrin on D3 (*p* = 0.0155) and filaggrin 2 on D3 (*p* = 0.0038) and D10 (*p* < 0.0001)]. Atopic samples showed increased epidermal thickness after allergen exposure (D3 vs. D0, *p* = 0.005), while normal dogs did not. In atopic samples, significant increased gene expression was found for filaggrin overtime but not for filaggrin 2. Western blot showed an increase in filaggrin 2 on D3. A small size band (15 kD) containing a filaggrin sequence was found in Western blots of atopic samples only. We conclude that atopic skin reacts to allergen exposure by proliferating and increasing filaggrin production but that it also has more extensive filaggrin degradation compared to normal skin.

## 1. Introduction

Atopic Dermatitis (AD) is a multifactorial disease resulting from a combination of genetic and environmental factors. Skin barrier impairment plays a role in both human and canine AD [1,2]. Filaggrin mutations are one of the most documented risk factors for AD in people [3,4]. Despite this strong association, the exact relationship between filaggrin alterations and disease development is incompletely understood. About 50% of AD patients carry loss-of-function mutations in filaggrin [5], but AD can occur in the absence of filaggrin mutation and the relevance of filaggrin mutation seems to vary depending on geographical location and age at the onset of the disease [6,7].

Filaggrin is a member of the S100 fused-type protein family and has been known for its important role in the barrier function and hydration of the stratum corneum [8]. Filaggrin originates as a large, heavily phosphorylated precursor protein (pro-filaggrin) in the stratum granulosum. The large precursor is located within the keratohyaline granules. The N-terminal domain is cleaved from profilaggrin and translocates to the nucleus where it is postulated to have a role in regulating terminal differentiation. This nuclear translocation is considered important for nuclear dissolution in the process of keratinization. Pro-filaggrin is dephosphorylated and cleaved into monomers. These monomers bind to keratin intermediate filaments, which leads to the collapse of the cytoplasm and the flattening of the cells into corneocytes. As corneocytes move outward in the stratum corneum, filaggrin is degraded further into natural moisturizing factors, which are important to maintain the hydration of the stratum corneum.

Another filaggrin-type protein, named filaggrin-2 (FLG2), was described in people in 2009 [9]. Filaggrin 2 is also a member of the S100 family, with an EF-hand domain at the N-terminus followed by a large repetitive domain. Filaggrin 2 appears to have an overlapping distribution as filaggrin and seems to also be important for proper cornification and skin barrier function [9,10]. Filaggrin 2 has also been reported to be decreased in people with AD, particularly in lesional atopic skin when compared to healthy controls [11]. T helper 2 cytokines decrease the expression of filaggrin 2, as has been reported to filaggrin, showing that inflammation modulates filaggrin expression.

A few studies have been published on filaggrin in dogs and its potential relevance to AD. Decreased filaggrin immunostaining in atopic skin biopsy samples compared to normal dogs was described [12]. Gene expression was reported to be increased in atopic dogs compared to normal dogs by various authors [13,14], suggesting that the increased production is possibly an attempt to compensate for an increased metabolism [15]. Interestingly, the distribution of immunostaining for filaggrin in atopic dogs was reported to be “patchier” and less homogeneous compared to normal dogs [14]. When research was conducted to identify mutations in atopic dogs, five SNPs within filaggrin were associated with canine AD, but only in certain breeds from different locations [16].

As time progressed and we learned about the existence of more than one filaggrin-type protein, it was realized that some reports in veterinary medicine have described filaggrin [12,17,18], while others reported a filaggrin-like protein now known to be filaggrin 2 [14]. No study so far has concurrently investigated the antibodies produced against filaggrin and filaggrin 2 in the same set of skin biopsies. One recently published study from our group has reported on the immunofluorescent staining of both filaggrin and filaggrin 2 on primary cell cultures of keratinocytes [19]. This study showed some overlapping features but also some differences in the staining between filaggrin and filaggrin 2.

The aim of the current study is to describe the staining intensity, distribution and pattern of both filaggrins in normal and atopic dogs during the course of allergen exposure. The purpose was to see differences between the two groups and evaluate how the allergen stimulation would affect filaggrins expression. The research dogs used for this study belong to a colony of atopic beagles, which have been validated as models for AD [20] and are known to mimic both the spontaneously occurring human and canine disease.

## 2. Materials and Methods

All procedures of this study were approved by institutional Animal Care and Use committee (#201910763). Since the used animals were research animals and not privately owned animals, no signed consent form was applicable.

### 2.1. Animals

All dogs used for this study were research animals. The atopic research beagles belonged to a colony of dogs that spontaneously developed AD and were easily sensitized by the epicutaneous application of allergens [21,22]. A flare-up of AD could be elicited upon re-exposure to the allergen of choice while controlling other factors such as diet and other allergens. In this model of disease, skin biopsies can be taken at various time points before, during and at the end of an allergen challenge. This allows us to gain information on baseline non-lesional atopic skin and compare it to acute lesional skin and then post-lesional skin, after the end of allergen exposure. The normal dogs were research beagles with no history or evidence of skin disease.

### 2.2. Protocol for Allergen Exposure

In the present study, all dogs (normal and atopics) were exposed to the allergen (*Dermatophagoides farinae*) 3 days in a row (D1, D2 and D3), thus D3 represented the peak of allergen exposure. An allergen challenge with *D. farinae* pure culture (Greer, NC, USA) was carried out epicutaneously by applying 25 mg in an aqueous solution on the inguinal area of the dogs. No additional allergen challenges were carried out after D3 to allow the skin to heal. Day 10 was representative of post-inflammatory lesions, after allergen exposure had ended.

### 2.3. Skin Biopsies

**Figure 1:**
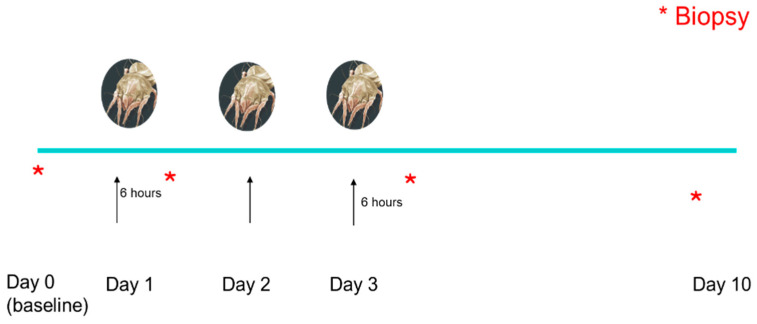
All dogs were challenged with the allergen three days in a row (Day 1, Day 2 and Day 3). Biopsies were taken prior to allergen exposure (Day 0), Day 1 (6 h after first allergen exposure), Day 3 (6 h after third allergen exposure) and on Day 10 (7 days after last allergen exposure). Arrows indicate times of allergen exposure. Red asterisks indicate times of skin biopsy.

Skin biopsies were taken prior to allergen exposure, on D0 (baseline), D1 (6 h after first allergen exposure), D3 (6 h after third allergen exposure) and on D10 (7 days after the last allergen exposure). In the atopic dogs, biopsies taken on D0 were from the non-lesional skin of the inguinal area, while biopsies after allergen challenges were taken from lesional areas. No lesions developed in the normal dogs as a result of allergen challenge at any time point and biopsies were taken from the areas where the allergen had been applied. Sequences of allergen challenges and biopsy collections are described in Figure 1.

One 8 mm punch tool was used to collect a skin biopsy from the inguinal area following a sub-cutaneous injection of 1 mL of lidocaine and sodium bicarbonate. All samples were divided and either processed for immunohistochemistry (IHC) by fixing in 10% neutral buffered formalin solution, and, after 48 h, being placed in saline until paraffin embedding, or immediately frozen in liquid nitrogen for extraction of RNA or protein for PCR and Western Blots.

### 2.4. Immunohistochemistry (IHC)

#### 2.4.1. Processing and Staining

Biopsies were embedded in paraffin, sectioned at 5 µm onto plus slides and dried overnight. Slides were deparaffinized and rehydrated using a series of xylene and EtOH washes. Antigen was retrieved using Tris-EDTA ph9, then enzymes were inhibited in 3% hydrogen peroxide in methanol. Remaining steps were performed using Lab Vision Autostainer 360. Biogenex reagents (power block, protein block) were used to block non-specific staining. Slides were incubated with primary antibody diluted with common antibody diluent from Biogenex for one hour at room temperature. Secondary antibody staining was performed using Biogenex Superenhancer (20 min) and Polymer-HRP (30 min). Washing steps were performed between incubations using PBS. Slides were incubated with Nova Red for 3 min and counterstained with hematoxylin for 10 s, dried overnight, then coverslipped with permount. The primary antibodies used had been previously validated and published in the literature. They were rabbit polyclonal antibodies for canine FLG [18] and canine FLG2 [14]. Control was normal rabbit serum.

#### 2.4.2. Image Analysis of IHC Staining

Five pictures of each biopsy section’s epithelium were taken using an EVOS microscope at 20X. Pictures were scored subjectively by four observers for intensity of staining (0–3), thickness of epithelium (0–3) and location of staining (Stratum Basale [SB], Stratum Spinosum [SS], Stratum Granulosum [SG], Stratum Corneum [SC]). Additionally, the staining was scored as either patchy or homogenous. The score for each picture was averaged between the three observers if the variable was an integer. If the variable was categorical, the percent of observers who indicated each category was calculated for each picture.

Pictures were also scored in a semiquantitative manner by using ImageJ (http://rsb.info.nih.gov/ij/, accessed on 1 June 2023). A pixel intensity threshold was determined to include only those image pixels in immunopositive areas [23]. The epidermis was traced and the total pixel count of the immunopositive area was calculated. The percentage of the immunostaining area was calculated by dividing the count of the immunopositive areas by the area of the epidermis.

### 2.5. Real-Time Polymerase Chain Reaction (RT-PCR)

RNA was extracted from frozen tissue using 5Prime PerfectPure RNA Cell Kit (cat no. FP2302500) following the kit’s protocol. Reverse transcriptase was performed using Super-script II (Invitrogen 18064-014, Waltham, MA, USA). Briefly, 1–3 µg RNA was added to a PCR tube with 1.5 µL 25 µM random hexamers (ThermoFisher N8080127, Waltham, MA, USA) and filled to 17 µL with DEPC H_2_O. This was heated to 70 °C for 10 min then placed on ice. The following was added to the tube: 6 µL 5× 1st Strand Buffer, 3 µL 0.1 M DTT, 1.5 µL 10 mM dNTP mix (ThermoFisher 10297018) and 1 µL Superscript II. This was heated to 25 °C for 10 min, 42 °C for 45 min, 95 °C for 3 min and kept at 4 °C until removal for use in real-time PCR. Real-Time-PCR was performed on StepOnePlus Real-Time PCR System (ThermoFisher 4376600) using PowerUp SYBR Green Master Mix (ThermoFisher A25741). Primers were diluted to 50 mM with DEPC H_2_O, then 10× primer mix was made using 100 µL each forward and re-verse primer and 800 µL of DEPC H_2_O. cDNA was diluted 1:2 before use in RT-PCR. Each well contained 12.5 µL 2× SYBR Green, 2.5 µL 10× primer mix, 2 µL cDNA and 8 µL DEPC H_2_O. We used the Quantitative ΔΔCt set-up on the StepOnePlus Real-Time PCR System. RPLO was used as housekeeping gene. Primers are as follows:

FLG1-GY:
  Forward 5′ GGGACACAAAGGGGATCCAG 3′
  Reverse 5′ CCAGACTTTCTGTGATGTTTTGTGA 3′
FLG2-GR:
  Forward 5′ AGATCGAGATCATGACCGAAGACT 3′
  Reverse 5′ CAGGCCATAGCCAGCTTGAA 3′
RPLO:
  Forward 5′ TTGTGGCTGCTGCTCCTGTG
  Reverse 5′ ATCCTCGTCCGATTCCTCCG

### 2.6. Protein Extraction from Skin for Western Blot

Protein extraction for Western blot analysis was achieved by using RIPA (radioimmunoprecipitation assay) buffer and HALT protease and phosphatase inhibitor cocktail (Thermos Scientific, Waltham, MA, USA) and 8 M UREA buffer were added. The skin biopsy was first homogenized using a Precelly tissue homogenizer with Bertin multibeads in 500 mls RIPA at 5000 rpm for 2 min, three times, placing on ice between bead beatings, then centrifuged at 12,000× *g* for 10 min at 4 °C. The supernatant was removed and 250 µL of the 8 M UREA buffer was added to the insoluble pellet and vortexed multiple times to dissolve. Samples were placed in −80 °C until further analysis and protein concentrations were determined by BCA assay.

### 2.7. Western Blot

Protein samples were electrophoresed using Novex^®^ NuPAGE^®^ 4–12% Bis-Tris gel (Product # NP0321BOX), XCell SureLock™ Electrophoresis System (Thermo Fisher Scientific, Waltham, MA, USA, Product # EI0002) and Precision Plus Protein™ WesternC™ Protein Standards (Bio-Rad, Hercules, CA, USA, Product # 1610376) at 120 volts for 1 h and 30 min. Proteins were then transferred onto a PVDF Membrane (Thermo Fisher Scientific, Waltham, MA, USA, Product #LC2002) using the XCell II™ Blot Module (Thermo Fisher Scientific, Waltham, MA, USA, Product #EI9051) at 280 mV for 1 h 30 min. The membrane was blocked for one hour with 5% Bovine Serum Albumin (BP1600-100) at 25 °C. The membrane was probed with the relevant primary antibody overnight at 4 °C followed by washes in PBST and HRP conjugate secondary antibody incubation (Amersham ECL Western Blotting Detection Kit RPN2108, 1:2000 dilution) for 1 h at 25 °C. Chemiluminescent detection was performed using Amersham ECL Western Blotting Detection Kit RPN2108 (Sigma-Aldrich, Inc, St. Louis, MO, USA). Densitometry analysis of western blot was performed using Azure densitometrics™ (Azure biosystems, Dublin, CA, USA).

### 2.8. Statistics

An average score for each biopsy section was used for the statistical analysis of IHC staining. Ten atopic dogs and five normal dogs were used for this study, but there were some missing points and the number of slides examined at various time points was not always the same. For example, for filaggrin, one atopic dog’s slides were missing on Day 0 due to technical issues and, on Day 3, one normal dog was not biopsied due to excessive bleeding in previous biopsies. Because of these missing values, a mixed-effects model (REML) was used in most analyses. For Filaggrin 2 analysis of only the atopics, where there were no missing values, a repeated measure one-way ANOVA was used. For the subsequent multiple comparisons, the two-stage linear step-up procedure of Benjamini, Krieger and Yekutieli was used. The correlations between subjective and semiquantitative staining were evaluated using both the Person’s product moment correlation (for linear correlations) and the Spearman’s rank correlation (for non-linear correlations). *p* < 0.05 was considered significant.

## 3. Results

### 3.1. Immunohistochemistry (IHC)

General observations: comparison of normal vs. atopic samples

**Figure 2:**
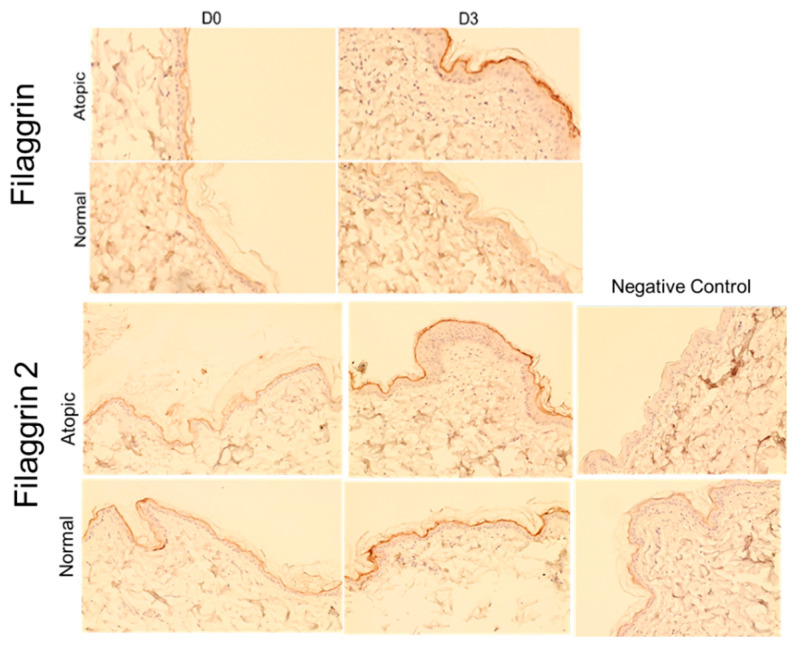
Example pictures of filaggrin and filaggrin 2 staining. Filaggrin intensity of staining in atopic skin biopsies is increased compared to normal dogs on Day 3, at the peak of allergen exposure (top row, right column). Filaggrin 2 staining intensity appeared increased after allergen challenge in both normal and atopic samples (second and third row, second images from the left). Epidermal thickness increased from Day 0 to Day 3 in atopic but not normal dogs (first and third row, Day 3)**.** Polyclonal antibodies produced in rabbits were used for the staining of both filaggrins. Negative control slides for both normal and atopic dogs are shown on the right column on the last two rows. For negative control, serum of rabbit prior to immunization was used. Pictures were taken at 10×.

Staining for both filaggrins was visible in the stratum granulosum and, to some degree, in the stratum corneum in both normal and atopic dogs (see Figure 2). Atopic dogs appeared to have a thicker epidermis and more intense filaggrin staining at the peak of the allergen challenge (Day 3, Figure 2).

Effect of allergen exposure in atopic samples

**Figure 3:**
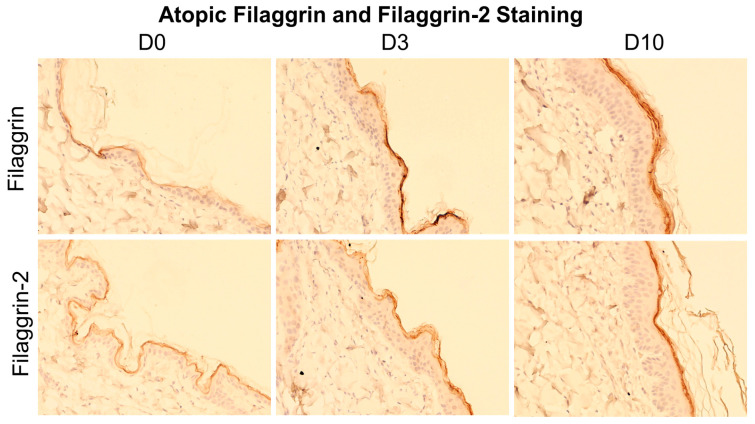
Example pictures of filaggrin and filaggrin-2 staining in one atopic dog on Days 0, 3 and 10. All pictures are from the same dog. Pictures were taken at 10×. Top row, IHC for filaggrin using a rabbit polyclonal antibody; bottom row, IHC for filaggrin 2 using a rabbit polyclonal antibody. Note the increased epidermal thickness on Day 10 compared to Day 0.

Within the atopic group, the intensity of staining for filaggrin and filaggrin 2 over the course of the challenge increased, with Days 3 and 10 showing an increased thickness of the epidermis and more visible staining in the stratum granulosum (Figure 3).

#### 3.1.1. Subjective Scoring of Staining on IHC

- **Intensity** of filaggrins staining

**Figure 4:**
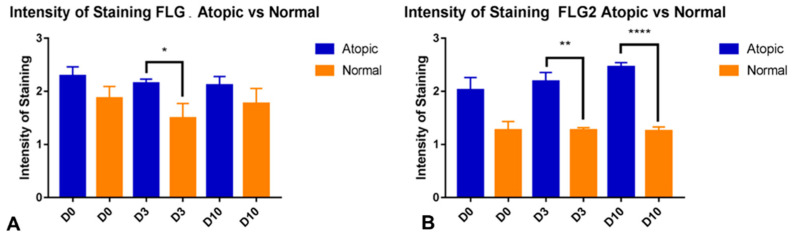
Subjective scoring of filaggrin intensity. Each slide was scored from 0 (no staining) to 3 (strong staining) by three people. This was averaged for each picture, and then averaged for each slide. (**A**): for filaggrin, on Day 3, atopic samples had higher intensity staining than normal samples (*p* = 0.0155). (**B**): for filaggrin 2, atopic samples had higher intensity of staining on both Day 3 (*p* = 0.0038) and Day 10 (*p* < 0.0001). The graphs show Mean and Standard Error of the Mean (SEM). Asterisks show significant comparisons. * for *p* < 0.05; ** for *p* < 0.005; **** for *p* < 0.0001.

When the intensity of filaggrin staining was scored subjectively by observers unaware of the source of the samples and the time points of biopsy collection, there were effects of group (*p* = 0.0134) and time (*p* = 0.0422). When the intensity of filaggrin 2 staining was scored subjectively, there was an effect of group (*p* = 0.0129) but not time. During subsequent multiple comparisons, there were no differences between normal and atopic samples at baseline for either filaggrin or filaggrin 2, but atopic samples had a higher intensity of staining than normal dogs for filaggrin on Day 3 (*p* = 0.0155, Figure 4A) and for filaggrin 2 on Day 3 (*p* = 0.0038) and Day 10 (*p* < 0.0001, Figure 4B).

b. **Epidermal thickness**

**Figure 5:**
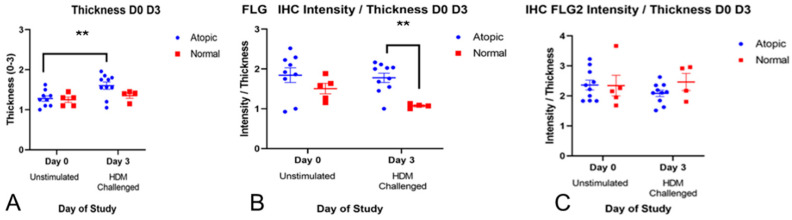
Epidermal thickness significantly increased in the atopic samples after allergen exposure ((**A**), *p* = 0.0048). When subjective scoring of intensity staining was divided by the thickness, atopic samples after challenge (Day 3) had a higher ratio than normal for filaggrin (*p* = 0.0048, (**B**)) but not for filaggrin 2 (**C**). ** indicates statistically significant difference (*p* < 0.005).

Epidermal thickness was also subjectively scored. The mixed-effect model (REML) showed the significant effect of day (*p* = 0.0287). The two-stage linear step-up procedure of Benjamini, Krieger and Yekutieli showed a significant increase in thickness in the atopic group (*p* = 0.0048) on Day 3 (Figure 5A). When the subjective scoring of staining intensity was divided by the thickness, atopic samples on Day 3 had a higher ratio than normal dogs for filaggrin (*p* = 0.0048, Figure 5B) but not for filaggrin 2 (Figure 5C).

c. **Homogeneity vs. patchiness of staining**

**Figure 6:**
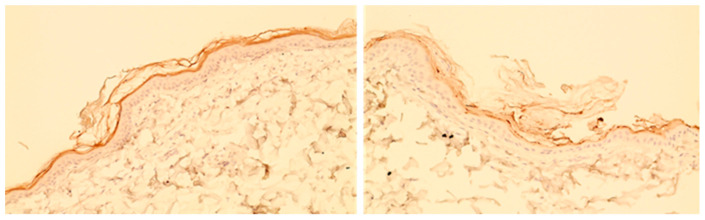
Examples of continuous staining (picture on the (**left**)) and patchy staining (picture on the (**right**)).

Immunohistochemical staining was subjectively scored for homogeneity. Staining could be classified as either homogeneous or patchy (see Figure 6 for examples).

**Figure 7:**
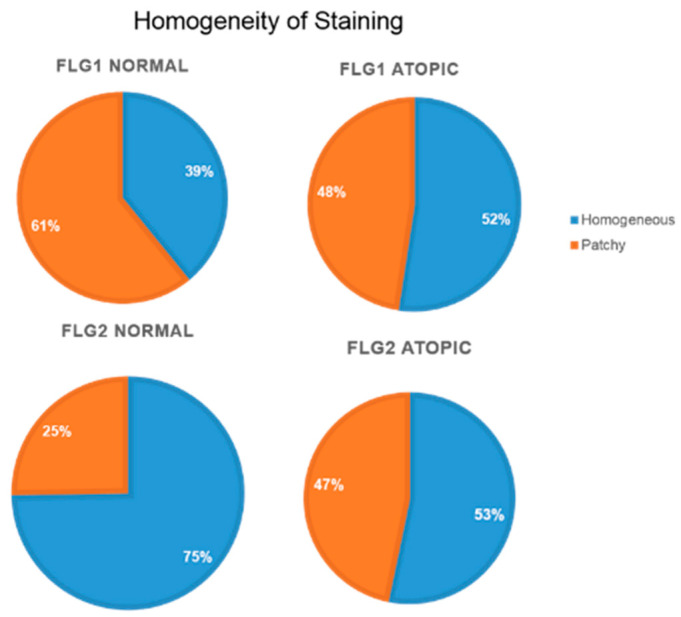
Percentages of pictures in the normal and atopic samples that were classified as either patchy or homogeneous, all times combined.

Figure 7 shows the percentage of pictures in the normal and atopic samples that were classified as either patchy or homogeneous. In the atopic samples, approximately half were considered to show homogeneous staining for both filaggrin and filaggrin 2, while, for the normal samples, only 39% were considered homogeneous for filaggrin and 75% homogeneous for filaggrin 2.

**Figure 8:**
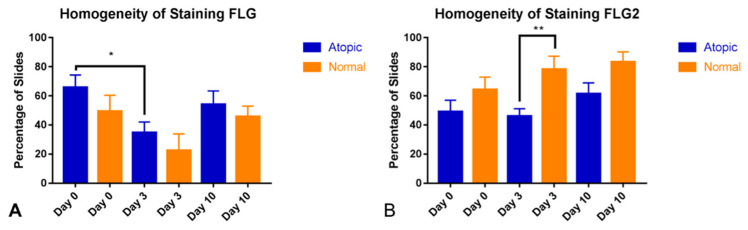
Homogeneity of filaggrin staining significantly decreased as a consequence of allergen challenge in atopic samples (Day 3 vs. Day 0, *p* = 0.0124, (**A**)). For filaggrin 2, no significant effect of time was detected but homogeneity was significantly less in atopic samples compared to normal samples on Day 3 (*p* = 0.0047, (**B**)). * indicates p less than 0.05; ** indicates p less than 0.005.

In the atopic dogs, the homogeneity of filaggrin staining decreased as a consequence of the allergen challenge (significant decrease on D3 compared to D0, *p* = 0.0124, Figure 8A). Although no significant changes in homogeneity of filaggrin 2 staining were observed as an effect of the allergen challenge, normal samples scored higher than the atopic samples on Day 3 (*p* = 0.0047, Figure 8B)

#### 3.1.2. Semiquantitative Scoring of Staining Intensity (Image J)

**Figure 9:**
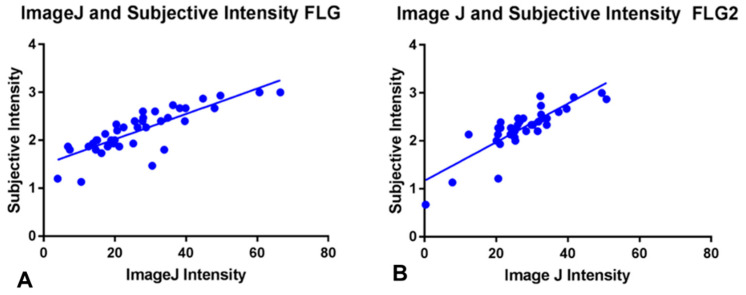
Positive significant correlations were found between the subjective scores for intensity of staining and the semiquantitative scoring using Image J for both filaggrin (**A**), Pearson r: 0.8189, r^2^: 0.6706; *p* < 0.0001) and filaggrin 2 (**B**), Pearson r: 0.8405, r^2^: 0.7064; *p* < 0.0001).

Besides the subjective scoring of the intensity of IHC staining, a semiquantitative assessment was also carried out using Image J. The correlations between subjective and semiquantitative (Image J) staining measurements were analyzed and positive significant correlations were found for both filaggrin (Pearson r: 0.8189, r^2^: 0.6706; *p* < 0.0001, Figure 9A) and filaggrin 2 (Pearson r: 0.8405, r^2^: 0.7064; *p* < 0.0001, Figure 9B).

### 3.2. PCR

**Figure 10:**
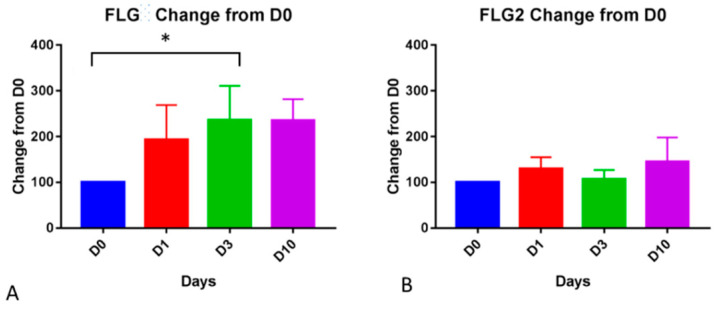
Changes from baseline in gene expression of filaggrin (**A**) and filaggrin 2 (**B**) in atopic samples during the course of the allergen challenge. These graphs show Mean and Standard Error of the Mean. The change in expression from Day 0 to Day 3 was significant for filaggrin (*p* = 0.01). No other significant changes were found. * indicates *p* value less than 0.05.

The gene expression of filaggrins was measured in atopic samples by PCR. A One-Way Repeated Measure ANOVA showed the significant effect of time for filaggrin (*p* = 0.043). When the gene expression of filaggrin was compared to the baseline, D3 was significantly higher (*p* = 0.01, Figure 10A). No significant changes were found for filaggrin 2 gene expression overtime (Figure 10B)

### 3.3. Western Blot

**Figure 11:**
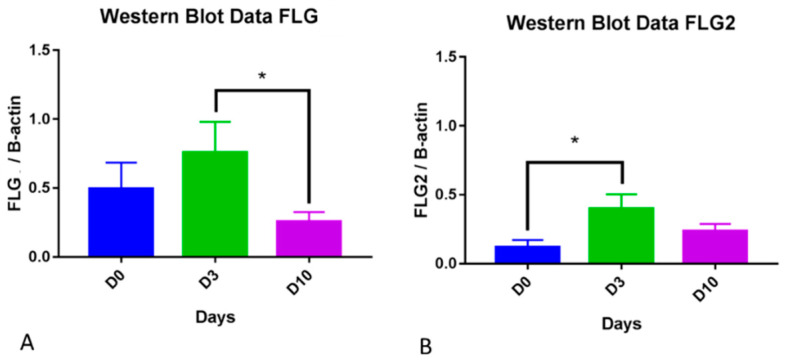
Filaggrin and filaggrin 2 protein expression during the course of allergen challenge. For filaggrin, a significant decrease was noted on Day 10 compared to Day 3 (*p* = 0.04), (**A**)). For filaggrin 2, a significant increase was seen on Day 3 compared to Day 0 (*p* = 0.0201, (**B**)). * indicates *p* values less than 0.05.

For filaggrin, the One-Way Repeated Measure ANOVA approached significance (*p* = 0.055). Upon further comparisons, filaggrin significantly decreased on D10 compared to D3 (*p* = 0.04; Figure 11A). For filaggrin 2, Friedman’s test showed significance (*p* = 0.0158) and Dunn’s Multiple Comparison showed higher filaggrin 2 on D3 compared to D0 (*p* = 0.0201, Figure 11B).

**Figure 12:**
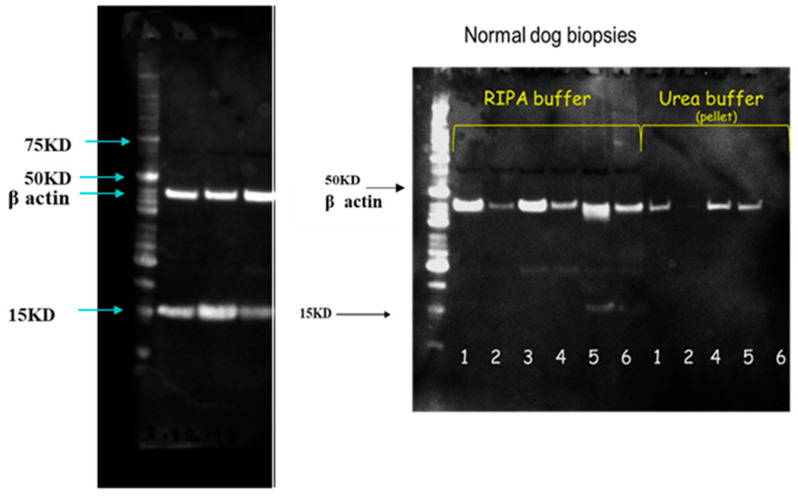
On the left, representative Western blotting probed with anti-dog filaggrin antibody in protein extraction from biopsies of an atopic dog biopsied on day 0, 3 and 10 of the allergen challenge (RIPA buffer). On the left side of each blots there is the ladder. The band at 15 kD was sequenced and was confirmed to be a part of filaggrin. This band was a consistent finding in atopic samples. On the right, representative Western blotting probed with anti-dog filaggrin antibody in protein extraction from biopsies of normal dogs taken on Day 0. The band at 15 kD was not seen in most normal dogs and is faint in dog 5 (RIPA buffer).

Western blots probed with anti-canine filaggrin antibody showed a band around 15 kD in atopic skin samples only (Figure 12, left picture). This finding was consistent for atopic samples. The band was sequenced and confirmed to be a part of filaggrin. This band was not detected in normal dogs (Figure 12, right picture).

**Figure 13:**
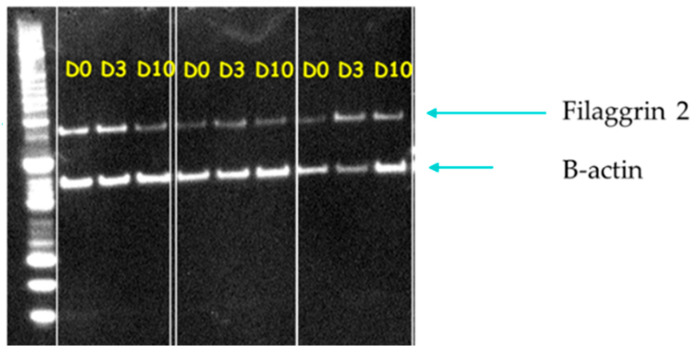
Representative Western blotting probed with anti-dog filaggrin 2 antibody (protein extraction of pellet with urea) from biopsies of three atopic dogs taken on Day 0 (D0), Day 3 (D3) and Day 10 (D10). On the left side there is the ladder.

Western blots for filaggrin 2 showed a consistent band around 54 kD (Figure 13).

## 4. Discussion

In this study, we described the expression of two filaggrin-like proteins in biopsies taken from atopic and normal beagles challenged with dust mites. The antibodies that we used for our studies had been validated and used in previous publications. The one for filaggrin had been used by Kanda et al. [18] and that for filaggrin 2 had been used by Santoro et al. [14].

**Figure 14:**
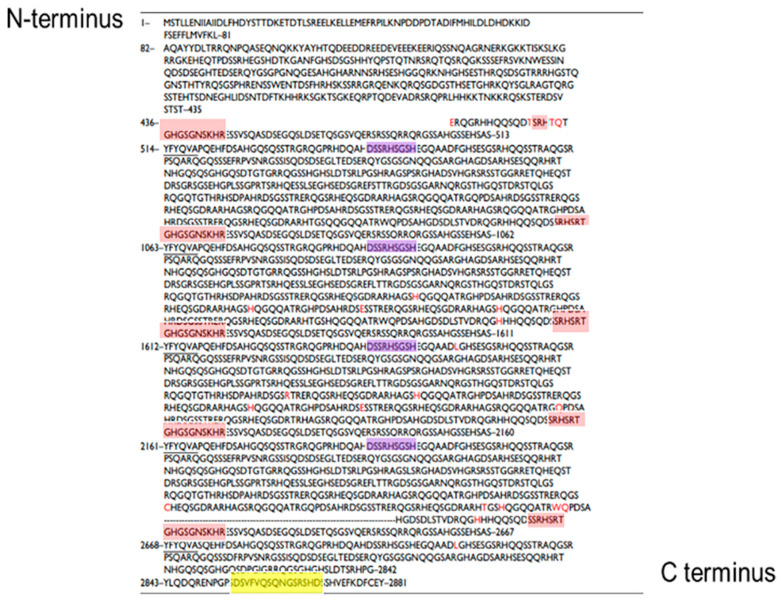
Sequence of canine filaggrin and summary of where various antibodies used for filaggrin are expected to bind the protein. Red highlight is the antibody used in our study (same as Kanda); purple and yellow highlights are Chervet’s antibodies.

When comparing the results of our studies with other publications, it is important to consider that different antibodies have been used by various authors. The one that we used for filaggrin was polyclonal and it was prepared through immunizing rabbits against a sequence which is located within the canine filaggrin monomer and canine pro-filaggrin [18] (see the red highlight in Figure 14 to visualize where this antibody binds onto filaggrin). Kanda et al.’s publication reported on IHC in normal canine skin but not in atopic skin. Kanda et al. described, like us, that filaggrin staining in normal canine skin was detected primarily in the stratum granulosum, but some was also visible in the corneum. Western Blotting probed with anti-dog filaggrin antibodies in Kanda’s report detected bands of 59 and 54 kD in normal skin, corresponding to the monomer repeats, which, in dogs, are of 549 and 507 amino acids. In the current study, we found bands around 54 kD in normal skin. Our study also used this specific antibody to stain atopic skin. In atopic skin, when membranes were probed with the same antibody, we also found a 15 kD band, which was not evident in normal skin. This band was sequenced and found to be part of filaggrin and was a consistent finding across the atopic samples.

Other authors have reported on filaggrin staining in canine atopic skin but they have used other antibodies. For example, Chervet et al. [12] used two antibodies for filaggrin when staining atopic skin samples. The N-terminal filaggrin antibody (ab24584, abcam, Cambridge, United Kingdom) was raised against DSQVHSGVQVEGRRGH of human filaggrin. The authors stated that this anti-human filaggrin antibody would recognize the discontinuous epitope DSSRHSGSH of canine filaggrin (see purple highlight in Figure 13). In Chervet’s study, the antibody against the C-terminal domain of canine filaggrin (DSV-42i and DSV-28i) was raised in rabbits against the C-terminal peptide DSVFVQSQNGSRSHD [12] (see yellow highlight in Figure 14). In normal dogs, Chervet et al. reported two distinct lines for both the N-terminal and the C-terminal filaggrin antibodies. In atopic dogs, using the C terminal antibody, they reported four different patterns. In our study, we did not use an antibody that targeted the C-terminal part of filaggrin, thus we cannot make any direct comparison between our findings and their findings.

Years ago, our group used an antibody raised against human filaggrin [24], N terminus (MSTLLENIFAIINLFKQYSKKDKNTDTLSKKELKELLEKEFRQILKNPDDPDMVDVFMDHLDIDHNKKIDFTEFLLMVFKLAQAYYESTRKENLPISGHK). This antibody cross-reacted with canine filaggrin but also showed staining in other epidermal proteins in the canine epidermis, as staining was also visible in the basal cell layers. It is important to note that canine and human pro-filaggrin sequences display little amino acid similarity (33%), except at the level of the S100 homologous part of the N-terminus (75%) [17,25]. The canine pro-filaggrin precursor predicted protein is approximately 317 kD and revealed four monomer repeats of 549 and 507 amino acids (fewer in number, but larger than in humans or mice) joined by linker sequences (YFY). The current study is an improvement compared to our previous report, as we used an antibody raised against canine filaggrin rather than using an antibody that had been raised against human filaggrin, although that antibody was partly cross-reactive with the canine counterpart. In the present study, no staining was visible in the stratum basale, while staining was evident in the stratum granulosum and corneum.

In our current study, we found that, at baseline, the intensity of filaggrin staining was not different between normal and atopic dogs. After allergen exposure, the atopic samples showed higher subjective scores for intensity of staining of filaggrin. We wanted to double check that the subjective scoring of intensity would be confirmed by a semiquantitative assessment of the intensity of staining using Image J and we indeed found a very good correlation between the subjective and the Image J measurements for the intensity of staining.

We found that, in atopic samples, there was an increased epidermal thickness after allergen challenges, while the normal dogs’ epidermis did not change in thickness. This suggests that, in atopic dogs, the allergic inflammation stimulated epidermal proliferation and differentiation. In terms of the increased thickness of the epidermis in the atopic samples after allergen exposure, it is important to point out that keratinocyte activation and proliferation is a characteristic of AD [26,27,28] and is linked to T helper 2 cytokines like IL-4 and IL-13, which act as growth factors for keratinocytes and increase proliferation [28,29,30].

A proliferative response after allergen exposure was not observed in normal skin biopsies in our study. The allergen challenge resulted in a decrease in staining homogeneity in the atopic samples compared to the normal dogs. The compensatory reactive response of atopic skin exposed to the allergen was also shown by the increased gene expression of filaggrin. This increased gene expression on Day 3 was not mirrored by a statistically increased amount of protein, as detected by Western Blot. This could either be due to the limited number of samples or be a reflection of the fact that, although cells are trying to compensate for an insult (allergen exposure), the fast degradation in atopic skin prevents an actual increase in the amount of protein. Filaggrin in atopic skin biopsies actually decreased after the allergen challenge was concluded (Day 10), returning to pre-challenge levels according to the Western Blot results. So, it is possible that the increased intensity of immunohistochemical staining for filaggrin on Day 10 is more a visual effect of a more prominent stratum granulosum rather than an actual increase in the amount of measurable protein.

The detection of the 15 kD band in blots probed with filaggrin antibody seen in atopic biopsies is of interest, as there are no prior reports of this band in the literature, possibly because Kanda only reported on normal skin biopsies and not on atopic samples. This was a consistent finding across the atopic samples. We sequenced the band, confirming that the protein had a sequence belonging to filaggrin; thus, this was indeed a specific finding. An explanation for this result only being present in atopic samples could be that atopic dogs had further degradation of filaggrin, which does not occur in normal individuals. The 15 kD band was also present in baseline biopsy samples, so it was an atopic feature and not the result of allergen exposure. Interestingly, when our group worked on the Western blots of filaggrin from cell cultures of atopic keratinocytes [19], this band was not detected. Thus, it is theorized that the additional degradation of filaggrin that occurs in skin biopsies of atopic dogs, and not in atopic keratinocytes grown in culture, may be the result of the cytokine milieu present in atopic skin biopsies and not an intrinsic feature of atopic keratinocytes. An increased degradation of filaggrin has been reported before in atopic skin biopsies [15]. More specifically, a significantly higher expression of calpain-1, caspase 14 and matriptase was found in atopic samples compared to control dogs, suggesting an increased breakdown of filaggrin in canine atopic skin. This increased breakdown of filaggrin in atopic dogs is similar to that which has been reported in mice models for AD [31] and contrasts with that which has been reported in human AD, for which a down-regulation of caspase has been reported [32,33] and decrease of filaggrin bioproducts [34,35].

It is currently accepted that filaggrin and filaggrin 2 have similar and overlapping functions and behaviors [36,37,38,39,40]. In our study, we found some similarities and some differences between filaggrin and filaggrin 2. Filaggrin 2 was more frequently scored as homogeneous in the normal samples compared to the atopic samples (75% vs. 53%). The patchiness seen in atopic samples is in line with previous reports by Santoro et al. [14], who commented on the patchy pattern of their immunofluorescent staining in atopic samples as the result of allergen exposure. The subjective scores for filaggrin 2 staining in atopic samples were higher than those of normal dogs as a result of allergen exposure, again possibly as a compensatory mechanism of atopic skin. Increased filaggrin 2 was confirmed in our Western blots at the peak of the allergen challenge (Day 3) in atopic skin samples.

In 2013, Santoro et al. produced canine-specific Abs in the rabbit from a synthesized immunogenic epitope to the predicted FLG2 protein [14]. The sequence of filaggrin 2 used for Santoro’s antibody production was GRRESSVTESSDTEND, which is not part of filaggrin’s sequence. To the best of our knowledge, this is the only published antibody used for canine filaggrin 2, thus we cannot compare our results to other publications besides Santoro’s. Immunofluorescent staining in Santoro’s report showed a homogenous distribution in the stratum granulosum and stratum corneum in healthy dogs and a patchy pattern in atopic dogs. We found similar results with samples from normal dogs scoring higher for homogeneity than the atopic samples. Using Western blot, a protein around 54 kD was detected with canine filaggrin 2 antibody. We found an increased staining intensity of filaggrin 2 in atopic samples compared to normal dogs on days 3 and 10. This is a different finding from Santoro, who reported increased gene expression in atopic vs. normal dogs as a result of an allergen challenge, but not an increase in the intensity of immunofluorescence staining at the peak of the allergen challenge (Day 3). Considering that dogs of the same colony were used for both studies and the same antibody was implemented, the reasons for these discrepancies are not clear. Both studies had a limited number of dogs, so it is important to further evaluate this behavior in larger studies. Notably, Schamber et al. using beagles from another colony and reported that filaggrin 2 gene expression and not filaggrin was decreased in the allergen-exposed skin of sensitized dogs [32]. This study also had a low number of allergic dogs (*n* = 6). It is possible that all these studies are largely affected by the small size and that, one day, when we have larger studies using dogs of multiple breeds with spontaneous AD and dogs with inflammatory diseases of other causes besides AD, a clearer picture on how filaggrin behaves is going to crystallize.

It is important to point out that the atopic dogs used for this study were dogs with proven familial predisposition to the atopic trait based on multiple breedings over the years. When puppies belonging to this colony were adopted out young, they were reported to spontaneously develop AD as private pets, even if they had never been sensitized when they were research dogs. Thus, these dogs were different from other models published in the literature, which typically utilize normal dogs to create a model of acute inflammation [41,42,43,44,45,46] or pruritus [47,48].

The dogs of our colony were known to have skin barrier abnormalities [49] and were the progenies of high IgE responders [50]; thus, they are, to the best of our knowledge, the closest representation of naturally occurring AD. Epicutaneous allergen exposure was used as a tool to time reactions and clinical flare-ups.

To conclude, we can state that allergen exposure in our atopic colony leads to a proliferative reaction. This was demonstrated by the increased epidermal thickness and the effort to proliferate and differentiate. We can also say that filaggrin degradation in the skin biopsies of atopic dogs appears more extensive than in normal dogs, as we found a band using Western Blot which was a specific finding in atopic skin samples and was not seen in normal biopsies. The finding of the 15 kD band is a novel finding in the literature. We also conclude that staining using an antibody for canine filaggrin and one for canine filaggrin 2 yields a similar epidermal location, suggesting the similar distribution of these two proteins.

## Data Availability

Data will be made available upon reasonable request.
